# A new genus of giant salamander (Urodela, Cryptobranchidae) from the Pliocene of Japan

**DOI:** 10.7717/peerj.21362

**Published:** 2026-06-03

**Authors:** Masahiro Noda, Masafumi Matsui, Kanto Nishikawa

**Affiliations:** 1 Graduate School of Human and Environmental Studies, Kyoto University, Kyoto, Japan; 2 Graduate School of Global Environmental Studies, Kyoto University, Kyoto, Japan; 3 The Geopark Research Centre, Institute for Environment and Development, Universiti Kebangsaan Malaysia, UKM Bangi, Malaysia; 4 Department of Biology, Faculty of Science, Chulalongkorn University, Bangkok, Thailand

**Keywords:** Cryptobranchidae, Pliocene, Kyushu Island, Japan

## Abstract

The family Cryptobranchidae, commonly known as giant salamanders, originated at least by the Late Paleocene and has persisted to the present day. However, its fossil record is, extremely fragmentary, with few occurrences known from East Asia after the Miocene. Here we report three vertebral specimens of a cryptobranchid salamander from the Upper Pliocene Tsubusagawa Formation (around 3.5 Ma) of Oita Prefecture, Japan. These specimens were previously identified as *Andrias* sp., including extant species, but their taxonomic status remained unresolved. In this study, we re-examine the material and provide a more detailed description and attempt to resolve its taxonomic status. Our study demonstrates that it is a new taxon, *Limnospondylus ajimuensis* gen. et sp. nov., because it shows a unique combination of characters in the mid-trunk vertebra. This new giant salamander inhabited freshwater lacustrine environments and may have reached a total length of approximately 110 cm by around 18 years of age. The discovery of this new taxon helps to fill a significant gap in the Asian fossil record of this group. It also highlights the morphological and ecological diversity of Cryptobranchidae and provides essential implications for understanding their evolutionary history.

## Introduction

The family Cryptobranchidae (Fitzinger, 1826) includes the largest extant amphibian worldwide, reaching up to 1.8 m in total length. They are aquatic, and morphologically uniform with few modern representatives and a relatively poor fossil record (Syromyatnikova et al., 2024). Two extant genera are currently recognized: *Andrias* Tschudi, 1837, comprised of five extant species from East Asia, namely *A. japonicus* (Temminck, 1836) in Japan, *A. davidianus* (Blanchard, 1871), *A. sligoi* (Boulenger, 1924), *A. jiangxiensis* Chai et al., 2022, and *A. cheni* (Gong et al., 2023) in China; and *Cryptobranchus* Leuckart, 1821, represented by *C. alleganiensis* (Daudin, 1803) in eastern North America.

The oldest unequivocal fossil records of Cryptobranchidae known to date include *Aviturus exsecratus* Gubin, 1991 and *Ulanurus fractus* Gubin, 1991 from the Late Paleocene of Mongolia, and *Cryptobranchus saskatchewanensis* Naylor, 1981 from the Late Paleocene of Canada. However, the taxonomic validity of *U. fractus* is unclear, and its phylogenetic affinities are poorly understood; therefore, this species has been strongly suggested to be a junior synonym of *Aviturus exsecratus* (Vasilyan et al., 2013). Other fossil taxa include *Zaissanurus beliajevae* Chernov, 1959 from the Late Eocene–Oligocene of Kazakhstan, *Andrias matthewi* (Cook, 1917) from the Miocene of North America, *Andrias scheuchzeri* (Holl, 1831), which has a long stratigraphic range from the Oligocene to the Pliocene, and *Ukrainurus hypsognathus* Vasilyan et al., 2013, a basal taxon from the Late Miocene of Europe. In contrast, fossil records from East Asia, where most of the extant species are concentrated, are incredibly scarce, particularly after the Miocene (Noda et al., 2025).

The Pliocene Tsubusagawa Formation in Kyushu Island, western Japan, has yielded exceptionally abundant vertebrate and invertebrate fossils (*e.g.*, Matsuoka & Kitabayashi, 2001; Takahashi & Kitabayashi, 2001; Iijima, Takahashi & Kobayashi, 2016; Handa et al., 2023). This assemblage is referred to as the Ajimu fauna (Takahashi, 2001). From this Ajimu fauna, fragmentary fossils attributable to Cryptobranchidae were discovered. These specimens, consisting of three vertebrae, were recovered between 1995 and 1997 from the banks of the Fukaimi River, where the Tsubusagawa Formation is exposed. These specimens were initially reported as belonging to *Andrias* (Matsui et al., 2001), but due to the limited comparative information of other fossil taxa available at the time, taxonomic identification could not be further refined. Because any fossil Cryptobranchidae from East Asia is crucial in the study of the evolution and biogeography of this group, we provide a detailed description of the Ajimu specimens and reassess their taxonomic position within Cryptobranchidae.

## Materials & Methods

**Figure 1:**
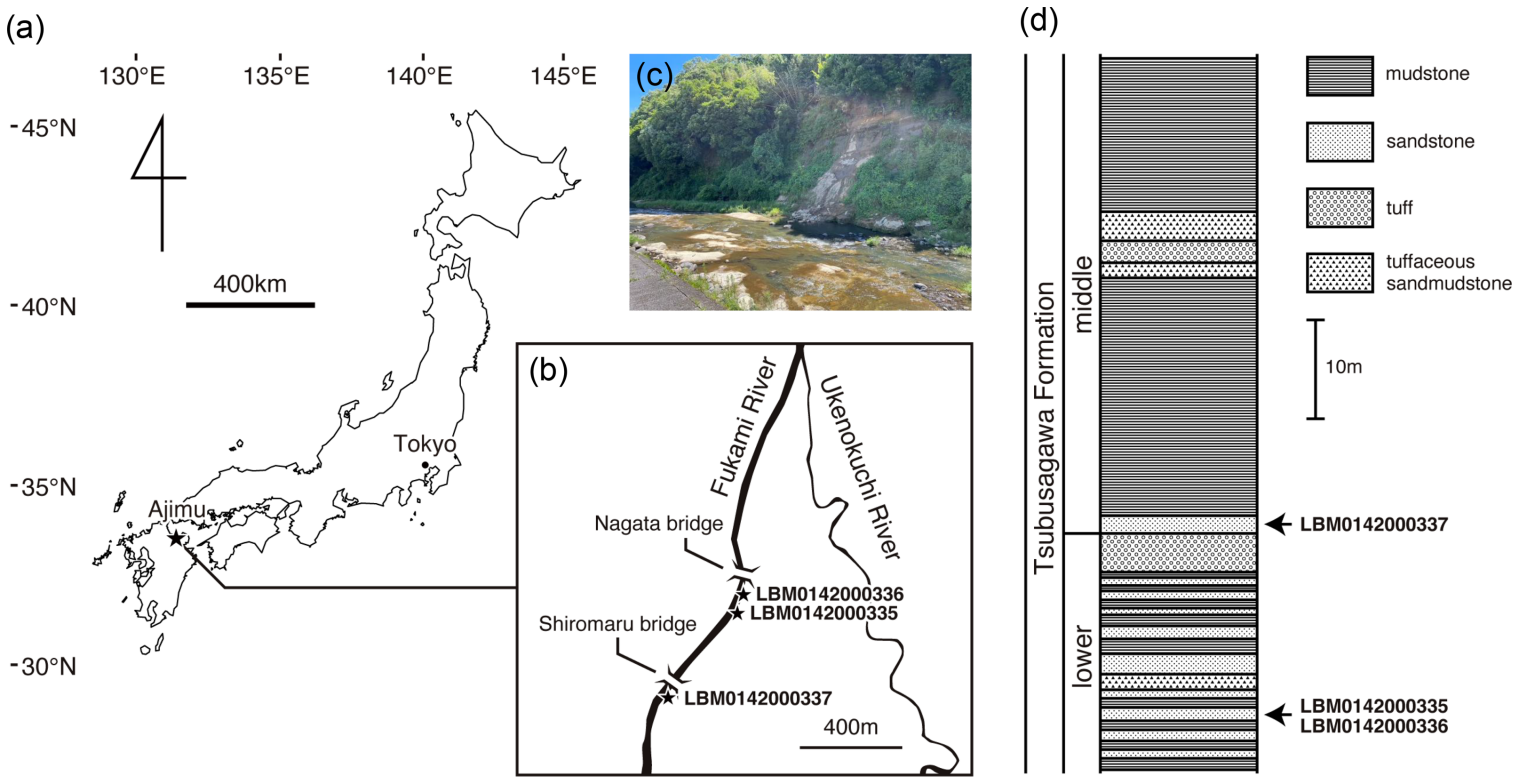
Geographic and geological context of giant salamander specimens from Ajimu. (A) Map of Japan showing the location of the Ajimu area in Oita Prefecture, Kyushu Island (indicated by a star). (B) Locality of *Limnospondylus ajimuensis* gen. et sp. nov. in the Ajimu area (indicated by a star). (C) Banks of the Fukami River, where the Ajimu fauna was discovered (photographed in 2024). (D) Stratigraphic column of the Tsubusagawa Formation exposed along the Fukami River, modified from Iijima, Takahashi & Kobayashi (2016).

Isolated three vertebrae attributable to Cryptobranchidae, analyzed in this study, were discovered by Mr. Eiichi Kitabayashi from the banks of the Fukami River in Ajimu-machi, Usa, Oita Prefecture, western Japan (Fig. 1). The first specimen (LBM0142000335) was collected in December 1995 from the right bank upstream of Nagata Bridge, corresponding to the lower part of the Tsubusagawa Formation. The second specimen (LBM0142000336) was recovered in August 1996 from a sandbank in the same area, also referable to the lower part of the formation. The third specimen (LBM0142000337) was obtained in August 1997 from the right bank upstream of Shiromaru Bridge, near the basal horizon of the middle part of the formation. It is reasonable to assume that the three vertebrae represent different individuals; however, all specimens were collected from closely similar stratigraphic levels—around the boundary between the middle and lower Tsubusagawa Formation and from the lower part. Based on tephrochronology and fission-track ages, the sediments containing the fossils are estimated to be approximately 3.5 Ma in age (Late Pliocene) (Hase et al., 2001; Satoguchi, 2001; Satoguchi, 2016). Further details of the Tsubusagawa Formation are provided by Hase et al. (2001) and Iijima, Takahashi & Kobayashi (2016). The same stratigraphic level has yielded a wide variety of vertebrate fossils, including fishes (Cyprinidae and Bagridae), crocodylians (Alligatoridae and Crocodylidae), turtles (Platysternidae, Trionychidae, and Bataguridae), birds (Pelecaniformes, Falconiformes, Anseriformes, and Gruiformes), and mammals (Stegodontidae, Cervidae, Ursidae, and Rhinocerotidae) (Aoki, 2018; Hirayama, 2001; Kato, 2001; Matsuoka, 2001; Nakajima & Kitabayashi, 2001; Takahashi & Kitabayashi, 2001; Iijima, Takahashi & Kobayashi, 2016; Handa et al., 2023).

The three specimens of cryptobranchids from Ajimu (LBM0142000335, LBM0142000336, and LBM0142000337) are currently housed in the Lake Biwa Museum. We loaned and examined them in detail. To obtain information on their internal morphology and three-dimensional structures, micro-CT scans were conducted using a system installed at Voxel Works Co., Ltd., Tokyo, Japan. Scanning parameters were as follows: isotropic voxel size of 18.5 µm, voltage 100 kV, and current 150 µA.

Each specimen was compared with the vertebrae of both extant and fossil cryptobranchids from the Cenozoic. Comparisons with the extant species *Andrias japonicus* (KUHE38393, 66656, KUZ-KYB7218, JMC Digital CT Museum of Life No. 16) and *Cryptobranchus alleganiensis* (KUHE38389, UMUT-A1807, NSM-H14544), as well as with the fossil species *A. matthewi* (AMNH-FARB8336, 8650, 8652, 10500, 10506, 42912) and *C. saskatchewanensis* (UALVP14875), were based on direct observations of skeletal specimens and CT data. Comparisons with the fossil taxa *Aviturus exsecratus* (PIN4356), *Zaissanurus beliajevae* (ZSN-KK-1), *Ukrainurus hypsognathus* (NMNHK22-1699), and *A. scheuchzeri* (JAM201921, 201931) were based on published descriptions and photographic materials provided by colleagues. Hereafter, measurement variables are abbreviated as follows: total length (TL), trunk vertebral centrum length (CL), and centrum height (CH). Morphological terminology follows Vasilyan et al. (2013).

### Geometric morphometric analysis

To quantify variation in two-dimensional vertebral shape, the Ajimu specimens were compared with vertebrae of the two extant genera, *Andrias japonicus* (*N* = 4) and *Cryptobranchus alleganiensis* (*N* = 3), using the 4th, 12th, and 18th trunk vertebrae. The raw image data were imported into a PC, and tps format files were created using tpsUtil (version 1.78) (Rohlf, 2015). Labeling points were then assigned using tpsDig2 (version 2.31). Outlines of the vertebrae were digitized as 10 landmarks in the dorsal view and 12 landmarks in the lateral view. Next, generalized Procrustes analysis was performed using MorphoJ (Klingenberg, 2011), and the designated points were standardized by translation, rotation, and scaling. Principal component analysis (PCA) was conducted to evaluate morphological differences in both dorsal and lateral views of vertebrae.

### Estimation of body length

Quantitative estimates of TL of the fossils were obtained by applying regression equations derived from the relationship between CL and TL in the two extant species, *Andrias japonicus* and *Cryptobranchus alleganiensis*. These equations were generated by regressing the maximum centrum length of trunk vertebrae against the total length of each individual, with both variables log-transformed. The sample included four individuals of *A*. *japonicus*, from which 19–20 trunk vertebrae were examined, and three individuals of *C*. *alleganiensis*, from which 18 trunk vertebrae were examined. To account for positional variation along the trunk series, three separate regression models were constructed based on measurements of the 4th, 12th, and 18th trunk vertebrae.

### Estimation of age

Counting growth rings on the articular surfaces of vertebral zygapophyseal processes is one of the established methods for estimating the individual age of both extant and extinct tetrapods (Syromyatnikova et al., 2024). This approach was recently applied to *Aviturus exsecratus* (Skutschas et al., 2020). In the Ajimu specimens, we found such growth rings in our preliminary observation, thus examined and counted these rings.

### Ethics statement and permits

All experimental procedures in this study followed the experimental animal guidelines of Kyoto University (approval nos. 29-A-7 and 30-A-7). The present study was conducted under the permissions issued by the Japan Agency of Cultural Affairs to K. Nishikawa for research in Kyoto City (no. 420) and in Kyoto Prefecture (no. 710).

### Nomenclatural acts

The electronic version of this article in Portable Document Format (PDF) will represent a published work according to the International Commission on Zoological Nomenclature (ICZN), and hence the new names contained in the electronic version are effectively published under that Code from the electronic edition alone. This published work and the nomenclatural acts it contains have been registered in ZooBank, the online registration system for the ICZN. The ZooBank LSIDs (Life Science Identifiers) can be resolved and the associated information viewed through any standard web browser by appending the LSID to the prefix http://zoobank.org/. The LSID for this publication is: urn:lsid:zoobank.org:pub:D4514909-6F7F-4E66-9BA7-8A17113C6A4C. The online version of this work is archived and available from the following digital repositories: PeerJ, PubMed Central SCIE and CLOCKSS.

## Results

### Comparisons to fossil and extant taxa

**Figure 2:**
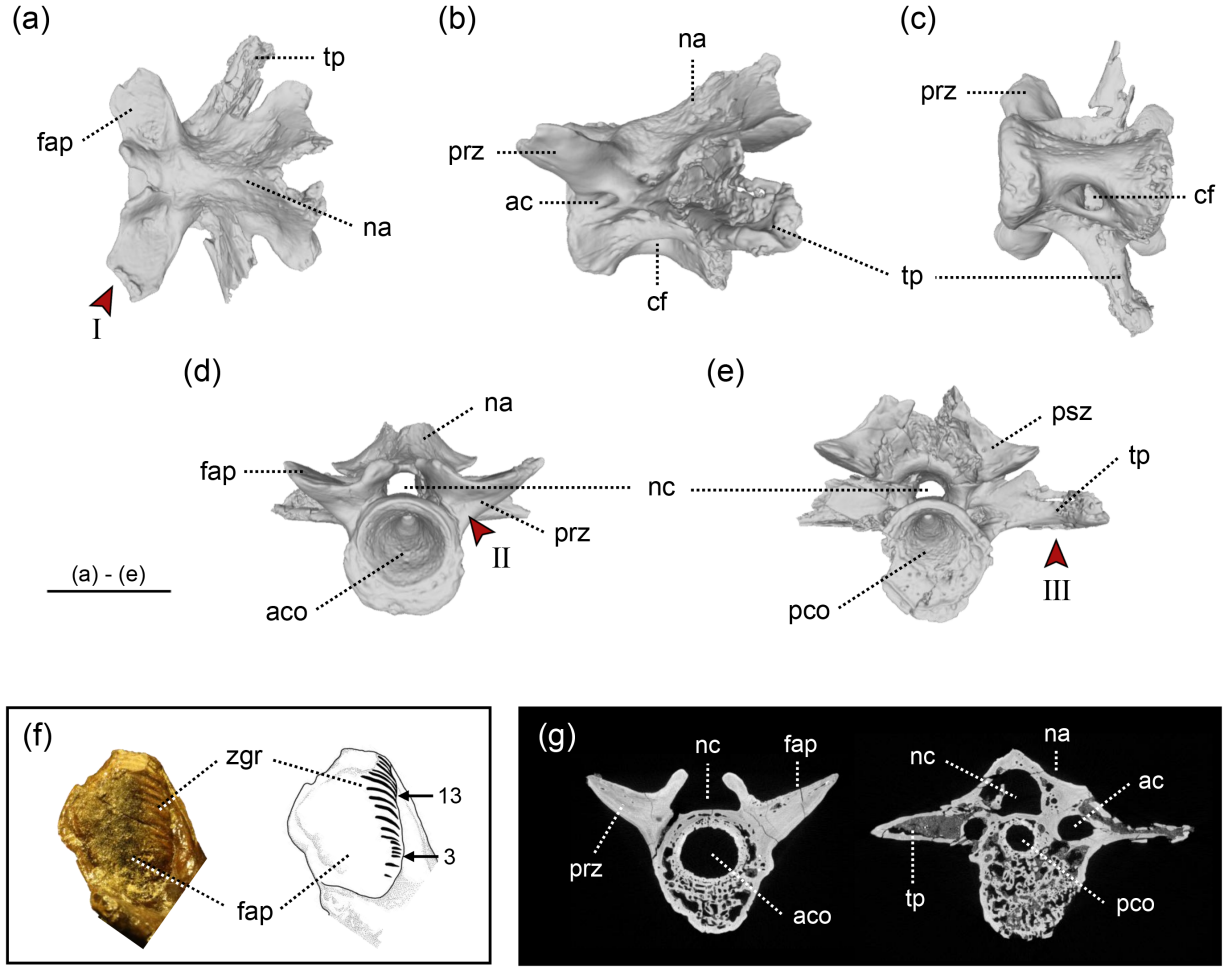
The holotype of *Limnospondylus ajimuensis* gen. et sp. nov. LBM0142000335 in dorsal (A), lateral (B), ventral (C), anterior (D) and posterior (E) views. (F) shows a close-up of the zygapophyseal articular surface (left) and an interpretative sketch (right). (G) shows CT scan images of cross-sections (left: anterior section; right: posterior section). Abbreviations: ac, anterior canal; aco, anterior cotyle; cf, central foramen; fap, facies articularis prezygapophysialis; na, neural arch; nc, neural canal; pco, posterior cotyle; psz, postzygapophysis; prz, prezygapophysis; tp, transverse process; zgr, zygapophyseal growth rings. Red arrows indicate the diagnostic characters: I. strongly laterally elongated prezygapophyses; II. broadly expanded and robust bases of the prezygapophyses; III. ventral margins of the left and right transverse processes form an almost straight line. Image b, shown in left lateral view, is horizontally flipped to show the morphology on the opposite side. Scale bar = 2 cm.

**Figure 3:**
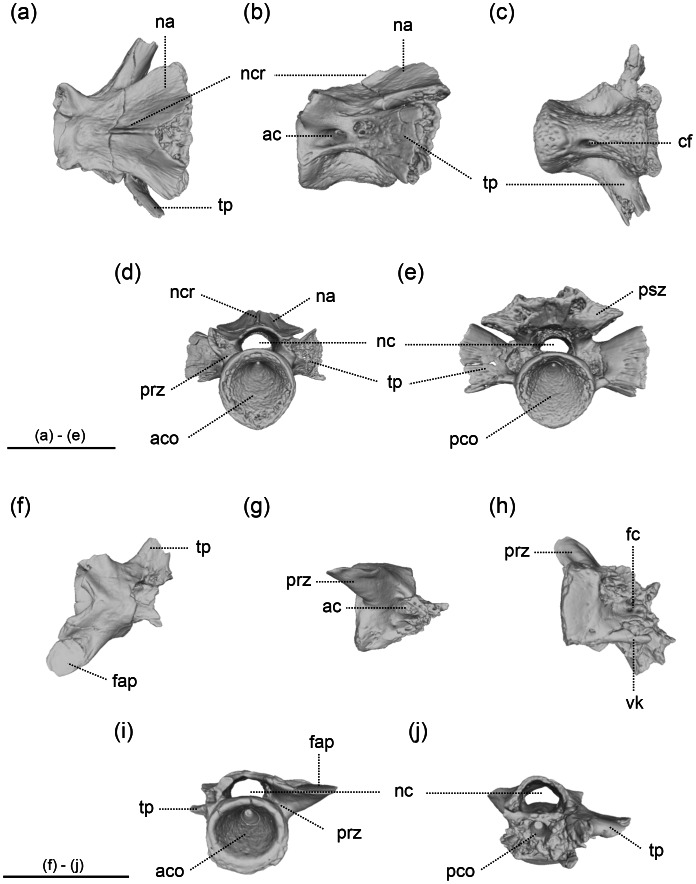
The vertebrae of *Limnospondylus ajimuensis* gen. et sp. nov. LBM0142000337 (A–E), LBM0142000336 (F–J) in dorsal (A, F), lateral (B, G), ventral (C, H), anterior (D, I) and posterior (E, J) views. Abbreviations: ac, anterior canal; aco, anterior cotyle; cf, central foramen; fap, facies articularis prezygapophysialis; fc, foramen centrale; na, neural arch; nc, neural canal; ncr, neural crest; pco, posterior cotyle; psz, postzygapophysis; prz, prezygapophysis; tp, transverse process; vk, ventral keel. Image b, shown in left lateral view, is horizontally flipped to show the morphology on the opposite side. Scale bars = 2 cm.

The three Ajimu specimens are represented by isolated vertebrae showing well-preserved external morphology (Figs. 2 and 3). The Ajimu specimens can be assigned to Cryptobranchoidea and Cryptobranchidae because of their large body size (see the estimation of body length in the next section), absence of the spinal nerve foramina in trunk vertebrae, and unicapitate transverse processes in trunk vertebrae (Westphal, 1958; Meszoely, 1966). Following the classification criteria of Gubin (1991), the Ajimu specimens could be referred to Aviturinae, characterized by vertebrae being rectangular, trapezoid, or parallelogram-shaped in profile, and prezygapophyses not projecting above the base of bony processes. However, the validity of these diagnostic criteria has been questioned by Vasilyan et al. (2013), who argued that most characters listed by Gubin (1991)—such as body size, dentary tooth count, vertebral shape, and some cranial or postcranial features—are highly variable, autapomorphic to *Aviturus*, or otherwise non-diagnostic for a clade-level definition.

**Figure 4:**
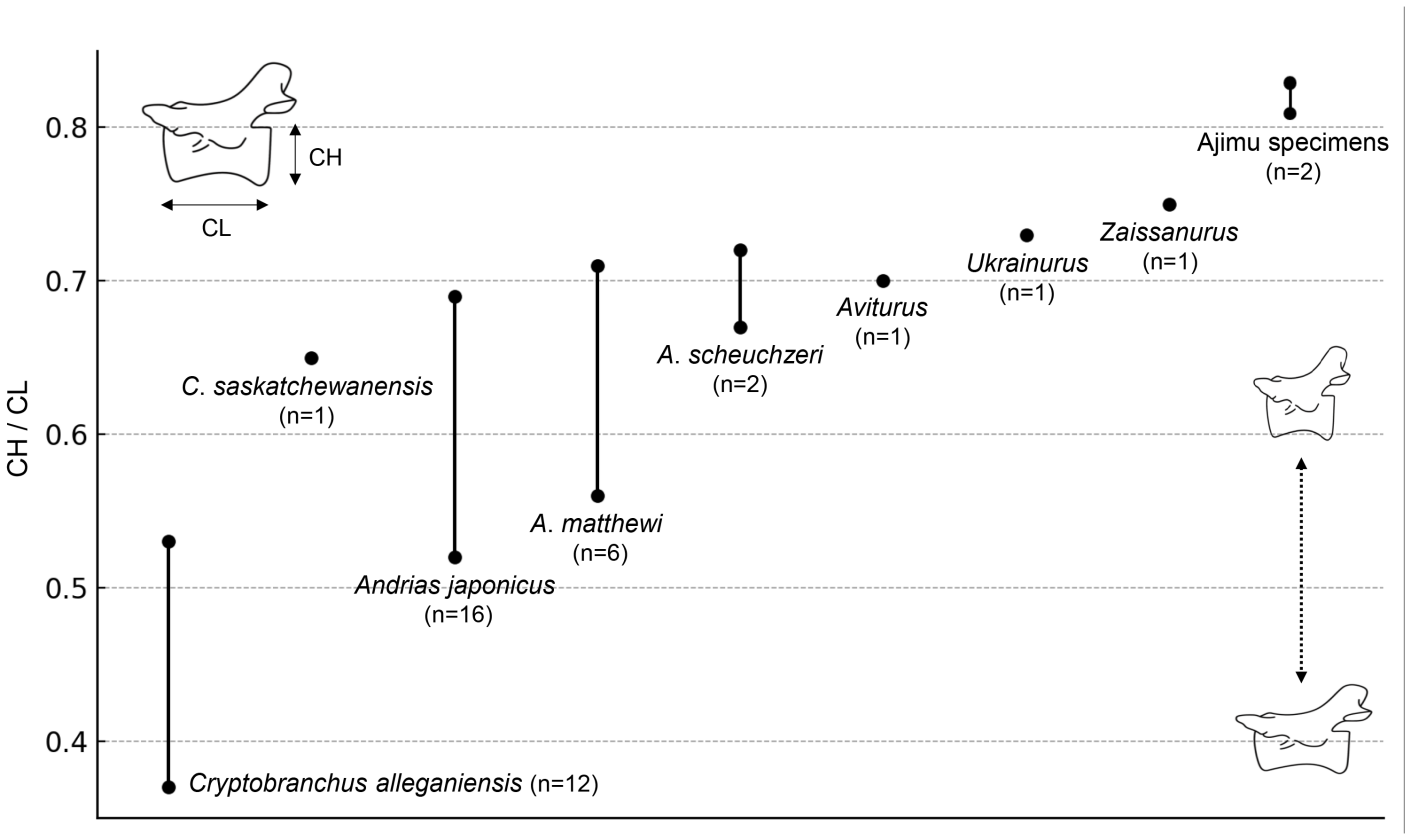
Ratio of centrum length (CL) to centrum height (CH) in extant and fossil cryptobranchid salamanders.

The results of the comparison of the ratio between CL and CH of Cenozoic cryptobranchids, including both extant and fossil species, indicated that the Ajimu specimens possess the shortest centra (Fig. 4).

In addition, the Ajimu specimen (LBM0142000335) exhibits characters that were not observed in all other cryptobranchids: in anterior view, broadly expanded and robust bases of the prezygapophyses; and in anterior and posterior views, the ventral margins of the left and right transverse processes together form a straight line.

**Figure 5:**
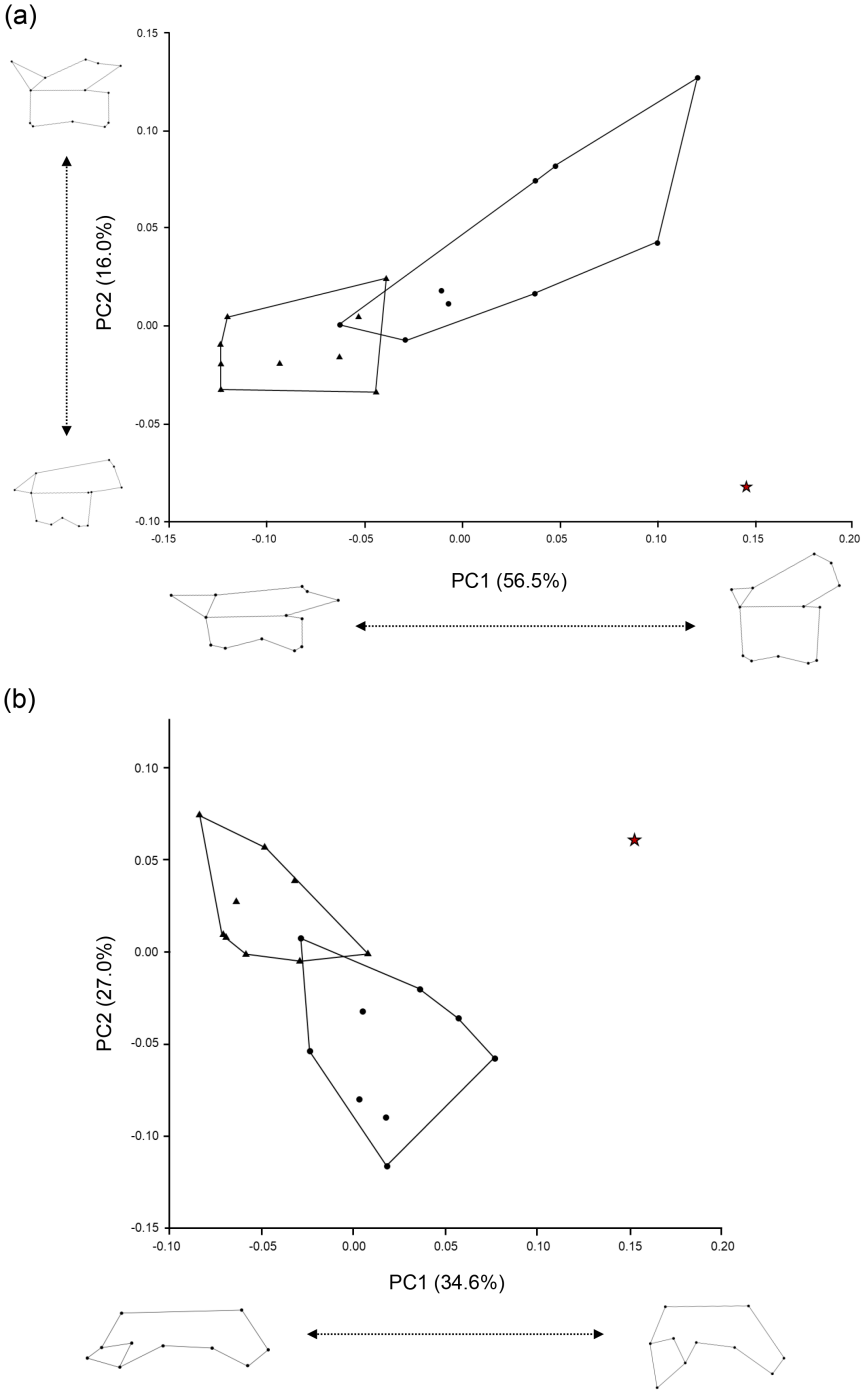
Scatter plots of PC1 and PC2 scores obtained from the geometric morphometric analysis. (A) Left lateral view of trunk vertebrae; (B) Dorsal half view of trunk vertebrae. The red star indicates the Ajimu specimen, circles indicate *Andrias japonicus*, and triangles indicate *Cryptobranchus alleganiensis*.

Furthermore, the results of the geometric morphometric analysis indicate that, in lateral view, the Ajimu specimen possesses centra that are shorter anteroposteriorly and taller dorsoventrally than those of the extant taxa *Andrias japonicus* and *Cryptobranchus alleganiensis*, and that the prezygapophyses do not project dorsally (Fig. 5A). In dorsal view, the prezygapophyses of the Ajimu specimen are markedly expanded laterally and form broad articular facets (Fig. 5B). The Ajimu specimen shares this character with *Aviturus exsecratus*, but it clearly differs from it by the absence of the interzygapophyseal ridge, which is an autapomorphic character of *Aviturus*.

### Body length

**Figure 6:**
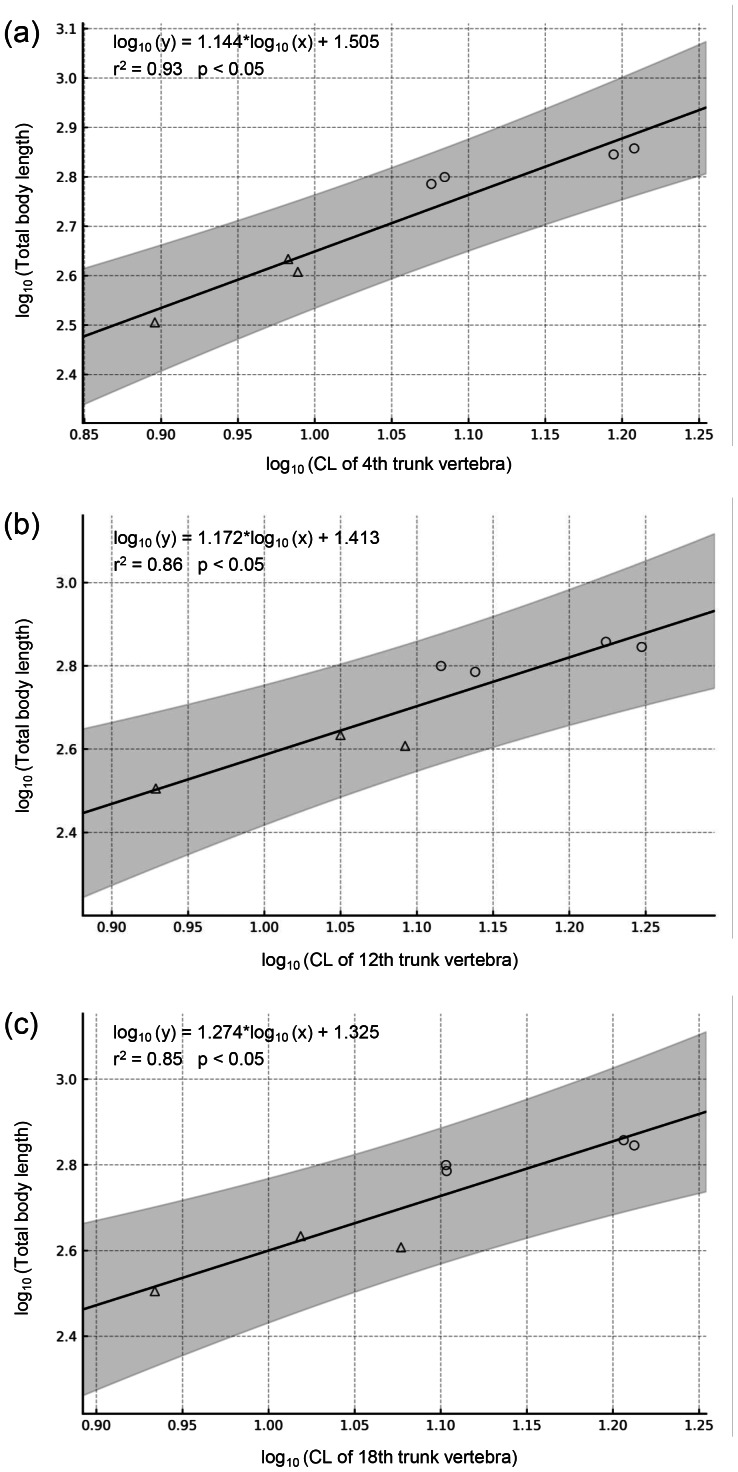
Regressions of vertebral metrics on total body length in two extant Cryptobranchidae. (A) Regression of centrum length of the 4th trunk vertebra on total body length (*p* = 0.0002). (B) Regression of centrum length of the 12th trunk vertebra on total body length (*p* = 0.0020). (C) Regression of centrum length of the 18th trunk vertebra on total body length (*p* = 0.0039). The gray band indicates the 95% confidence interval. Circles indicate *Andrias japonicus*, and triangles indicate *Cryptobranchus alleganiensis*.

Regression equations were derived based on vertebral measurements and total length of the two extant genera (Fig. 6). All regression models were statistically significant (*p* < 0.05) and had high explanatory power (*r*^2^ = 0.85–0.93), supporting their validity. Applying these models to the animal, the holotype and largest trunk vertebra (LBM0142000335) yielded an estimated TL of approximately 1,017.2–1,148.9 mm, with a prediction interval of 631.5–1,930.6 mm.

### Individual age

In the Ajimu specimens, the growth rings on the articular surfaces of vertebral zygapophyseal processes were observed only in the holotypic vertebra among the three specimens. Visually 17 or 18 zygapophyseal growth rings were observed on most of the articular surfaces of the zygapophyseal processes (Fig. 2F).

### Taxonomic position

The Ajimu specimen (LBM0142000335) can be clearly distinguished from extant and all other fossil cryptobranchids (extant *Andrias* spp., *A*. *scheuchzeri*, *A*. *matthewi*, *Cryptobranchus alleganiensis*, *C*. *saskatchewanensis*, *Zaissanurus beliajevae*, *Aviturus exsecratus*, and *Ukrainurus hypsognathus*) by a unique combination of vertebral features. Furthermore, the geometric morphometric analysis showed that the Ajimu specimen exhibits differentiated vertebral morphologies from those of the two extant genera, *A*. *japonicus* and *C*. *alleganiensis*. Notably, the degree of distinctiveness observed in the Ajimu specimen exceeds the variation found between these two extant genera, which are clearly distinguished from each other based on genetic data, external morphology, and osteological characters. From these lines of information, the Ajimu specimen cannot be accommodated within any known fossil or extant genus of Cryptobranchidae. Their distinctive morphology justifies recognition as a new genus, thus we described them as a new genus in below.

### Systematic paleontology

**Table:** 

Amphibia Linnaeus, 1758
Lissamphibia Haeckel, 1866
Caudata Scopoli, 1777
Cryptobranchidae Fitzinger, 1826
*Limnospondylus* gen. nov.

### Etymology

Greek limne (“lake”) and spondylos (“vertebra”), indicating the lacustrine habitat in which the animal occurred and the fossil vertebrae reported in this article.

### Type and only species

*Limnospondylus ajimuensis* sp. nov.

### Diagnosis

As for the type and only known species.

*Limnospondylus ajimuensis* sp. nov.

### Etymology

The specific name refers to Ajimu, the type locality of the new taxon.

### Holotype

LBM0142000335 (Fig. 2), a nearly complete mid-trunk vertebra preserving all diagnostic characters of the new taxon.

### Paratypes

LBM0142000336 (Figs. 3F and 3J), a caudosacral vertebra; LBM0142000337 (Figs. 3A and 3E), an anterior trunk vertebra. Both specimens are from nearly the same locality and horizon as the holotype and are referable to the new taxon.

### Repository

Lake Biwa Museum, Kusatsu City, Shiga Prefecture, Japan.

### Locality and horizon

The Fukami River, Ajimu-machi, Usa City, Oita Prefecture, Japan; upper Pliocene (approx. 3.5 Ma) Tsubusagawa Formation.

### Diagnosis

A cryptobranchid salamander diagnosable on the basis of the following combination of characters in mid-trunk vertebrae (autapomorphies marked by an asterisk): (1) anteroposteriorly short and dorsoventrally tall centra*; (2) in dorsal view, the prezygapophyses are strongly laterally elongated (shared with *Aviturus exsecratus*); (3) in anterior view, the bases of the prezygapophyses are broadly expanded and robust*; and (4) in anterior and posterior views, the ventral margins of the left and right transverse processes together form a straight line*.

### Description

The Ajimu specimens described in this study consist of three vertebrae (Figs. 2 and 3). The centra are deeply amphicoelous, anteroposteriorly short, and subtrapezoidal to parallelogram-shaped in lateral view, resembling an hourglass. In anterior and posterior views, the centra are nearly circular. The internal structure of each specimen indicates a low degree of bone compactness, with a porous architecture. In the original description, vertebral positions were determined solely by comparison with measurements of *A*. *japonicus*: LBM0142000335 as an anterior trunk vertebra, LBM0142000336 as a pre-sacral vertebra, and LBM0142000337 as a sacral vertebra. However, a more precise assessment of vertebral position requires qualitative evaluation of features such as ridges or keels and depressions that only measurements cannot capture. Accordingly, each specimen is redescribed in detail below, and its anatomical position reinterpreted by qualitative comparisons with complete skeletons of multiple individuals of *A*. *japonicus* and *C*. *alleganiensis*.

LBM0142000335 (Fig. 2) —This specimen is the largest and best preserved of the three vertebrae, lacking only the distal tips of the prezygapophyses, transverse processes, and neural spine. The prezygapophyses extend strongly laterally, with relatively large articular facets compared to those in LBM0142000336, and the articular surface is concave in cranial view. Growth rings are visible on the surface of the zygapophyseal articular facets. The neural arch arches strongly posteriorly from the mid-centrum, and the neural crest is not developed, unlike in LBM0142000337. The neural canal is relatively narrow compared to the other two specimens. Ventrally, a central foramen is present only on the right side, and the ventral surface of the centrum curves caudally in a hook-like fashion. The transverse processes extend nearly perpendicular to the centrum and project caudally, and an anterior canal perforates the bases of the prezygapophyses posteriorly. Measurements: CL (anteroposterior), 22.9 mm; CH (dorsoventral), 19.2 mm. Based on the combination of a deeply concave ventral midline of the centrum and the lack of a developed neural crest, this specimen is interpreted as a mid-trunk vertebra.

LBM0142000336 (Figs. 3F and 3J) —This specimen is heavily damaged, preserving only the anterior portion of the centrum, the left prezygapophysis, and the base of the right prezygapophysis. The posterior part of the centrum shows traces of strong compression, with deformation toward the sagittal axis, and localized carbonization is present. Although smaller than the other two specimens, it shares certain features, such as well-developed prezygapophyseal bases and a neural arch that exhibits slight posterior arching along the centrum. The prezygapophyses are strongly cranially directed, forming a V-shape, more pronounced than in the other specimens. The neural arch is largely absent, but the neural canal is the largest of the three specimens. A foramen centrale is present on the ventral surface of the centrum, with ventral keels observed on both sides. This feature is consistent with caudal or caudosacral vertebrae. Although the posterior half of the centrum is crushed and the presence of haemapophyses cannot be confirmed, no extension of haemapophyses is observed in the preserved mid-region of the centrum. Measurements: CL (preserved portion), 18.2 mm; CH, 14.1 mm. Based on the following combination of features, this specimen is interpreted as a caudosacral vertebra: prezygapophyses strongly directed cranially (V-shaped); the ventral surface of the centrum bearing a foramen centrale; and absence of haemapophyseal extension from the ventral midline.

LBM0142000337 (Figs. 3A and 3E)—This specimen is relatively well preserved, with only the tips of the neural spine and prezygapophyses broken. It is the only specimen in which the transverse processes are nearly completely preserved to their distal ends. The neural arch arches strongly posteriorly from the mid-centrum, and a well-developed neural crest with distinct ridge is present, unlike in LBM0142000335. The neural canal is broad, comparable to that of LBM0142000336. The ventral surface of the centrum bears a central foramen on the right side, as in LBM0142000335, but the curvature is weaker and the concavity shallower, giving the centrum a more box-like appearance. This morphology is characteristic of anterior trunk vertebrae in extant species. The transverse processes extend caudally, but are relatively short and expand dorsally in a fan-shaped manner, differing from those of LBM0142000335. Two or three ventral foramina for spinal nerves are present at the ventral base of the transverse process, and an anterior canal perforates the bases of the prezygapophyses posteriorly, as in LBM0142000335. Measurements: CL, 20.1 mm; CH, 16.3 mm. Based on the presence of a well-developed neural crest with a distinct ridge and the relatively shallow ventral concavity, this specimen is interpreted as an anterior trunk vertebra.

## Discussion

### Phylogenetic position

Phylogenetic analysis was not examined in this study. Cryptobranchids are well known for their morphological conservatism, and fossil material is often fragmentary, making it difficult to establish robust character matrices. Existing datasets (*e.g.*, Vasilyan et al., 2013) contain very few informative vertebral characters, and incorporating the new genus material would result in excessive missing data and unstable placements. Moreover, the validity of some higher-level groupings such as Aviturinae has already been questioned, reflecting the current instability of phylogenetic hypotheses within the family. Under these circumstances, a more responsible approach is to provide a detailed morphological description and comparisons, rather than forcing the material into an unreliable phylogenetic framework.

### Body length and age estimation

In this study, the total length of the individual with the largest centrum of the new genus was estimated to be 1,017.2–1,148.9 mm based on regression analysis of extant cryptobranchids (see Materials & Methods). However, because these models are derived from measurements of the two extant species, the estimates should be regarded as provisional until additional fossil material is discovered.

Growth rings observed on the articular surface of the prezygapophyses, although partly obscured by surface wear and damage, numbered at least 17. This suggests that the individual may have reached the estimated size at approximately 17 years of age. In *Aviturus exsecratus*, it has been reported that the spacing between growth rings narrows from the 5th–8th rings onward across seven examined specimens, which has been interpreted as indicating the attainment of sexual maturity at 50–60% of maximum body size (Skutschas et al., 2020). By contrast, in the new genus, the spacing between growth rings remains relatively uniform up to about the 13th ring, suggesting that the growth rate did not decline markedly during this interval.

### Paleoecology of the Ajimu giant salamander

During the Late Miocene–Pliocene, proto-Japan was connected to the Eurasian continent except during several marine transgression events around 3.5 Ma, 3.2 Ma, and 2.9 Ma (Kitamura & Kimoto, 2006; Takahashi & Izuho, 2012). The Ajimu fauna is closely related to the present-day fauna of the region extending from southern China to Southeast Asia (Takahashi, 2001; Takahashi & Izuho, 2012), suggesting that this fauna may have migrated from the continent. The Ajimu fauna includes large mammals such as the Japanese endemic proboscidean *Stegodon miensis* (Takahashi & Kitabayashi, 2001; Takahashi, 2018) and rhinocerotids (*e.g.*, Handa et al., 2023), as well as rodents of Palearctic origin (*Micromys* sp.; Kato & Kitabayashi, 2018) and elements of the Oriental realm, such as cervids (*Cervus unicolor*), crocodilians (*Alligator sinensis* and *Toyotamaphimeia machikanensis*), and turtles (*Platysternon megacephalum*, *Pelodiscus sinensis*, *Ocadia sinensis*, *Chinemys* cf. *nigricans*) (Hirayama, 2001; Takahashi & Kitabayashi, 2001; Iijima, Takahashi & Kobayashi, 2016; Aoki, 2018; Takahashi, 2018). Accordingly, the Ajimu fauna has been regarded as a subtropical to tropical assemblage that combined elements of both the Palearctic and Oriental realms (Hirayama, 2001), within which this giant salamander lived. The paleoenvironment of the Ajimu region at that time was characterized by extensive freshwater lakes, with wetlands spread across the surrounding lowlands and forests covering the margins (Hase et al., 2001). The new genus is therefore inferred to have inhabited freshwater lacustrine environments. Cryptobranchid salamanders occur in humid subtropical or temperate areas with temperatures of 8–25 °C, and their fossils can be useful palaeoclimatic and palaeoenvironmental indicators (Böhme, Vasilyan & Winklhofer, 2012). The new genus suggests favorable conditions for these animals in East Asia up to the Pliocene, *i.e.,* warm humid to very humid climates with mean annual precipitation exceeding 900 mm (Böhme, Vasilyan & Winklhofer, 2012). While the two extant genera are restricted to rivers, *i.e.,* lotic habitats, fossil records indicate that fossil cryptobranchids also inhabited standing-water environments such as lakes, as well as brackish habitats and even semiterrestrial settings (*e.g.*, Vasilyan & Böhme, 2012; Szentesi, Sebe & Szabó, 2019; Syromyatnikova et al., 2024). The new genus also may have wider niche than the extant species.

A recent molecular phylogenetic study indicates that the extant *Andrias* had already appeared by around 15.8 Ma in the Middle Miocene (Marr et al., 2024). Accordingly, this suggests that two cryptobranchid genera with different ecological adaptations inhabited East Asia around 3.5 Ma.

Climatic cooling during the Pliocene–Early Pleistocene transition (∼2.6 Ma) is known to have severely impacted herpetofaunal communities (Delfino, Rage & Rook, 2003). Such global environmental changes may have contributed to the extinction of the new genus. Some studies suggest that marine transgressions occurred in parts of western Japan during this period (*e.g.*, Kitamura & Kimoto, 2006; Iwatani, Irizuki & Goto, 2011), potentially reducing wetland and lacustrine habitats. However, it remains unclear whether such sea-level changes directly affected the Ajimu region. Regardless, the loss of freshwater habitats—whether due to climate cooling, local tectonic uplift, or any marine incursion—could have been a key factor in the disappearance of the new genus. In contrast, *Andrias japonicus*, already adapted to riverine environments, may have been less vulnerable to such habitat loss and survived to the present day. Interestingly, the Ajimu giant salamander was recovered from the Tsubusagawa Formation exposed along the Fukami River, where *A*. *japonicus* occurs today.

##  Supplemental Information

10.7717/peerj.21362/supp-1 Supplemental Information 1 Comparative character states of trunk vertebrae among cryptobranchid salamanders

10.7717/peerj.21362/supp-2 Supplemental Information 2 Measurements of trunk vertebrae of cryptobranchid salamanders

10.7717/peerj.21362/supp-3 Supplemental Information 3 Representative mid-trunk vertebrae (12th) of *Andrias japonicus* (a-c) and *Cryptobranchus alleganiensis* (d-f). Dorsal (a, d), anterior (b, e), and posterior (c, f) views. Scale bars = 20 mm

## Institutional abbreviations

AMNH: American Museum of Natural History, New York, USA
JAM: József Attila City Library and Museum Collection, Komló, Hungary
KUHE: Graduate School of Human and Environmental Studies, Kyoto University, Kyoto, Japan
KUZ: The Zoological Collection of Kyoto University, Kyoto, Japan
LBM: Lake Biwa Museum, Shiga, Japan
NMNHK: National Museum of Natural History, Kiev, Ukraine
NSM: National Museum of Nature and Science, Tokyo, Japan
PIN: Paleontological Institute, Russian Academy of Sciences, Moscow, Russia
UALVP: University of Alberta, Laboratory of Vertebrate Paleontology, Alberta, Canada
UMUT: The University Museum, The University of Tokyo, Tokyo, Japan
ZSN: Zaisan collection, National Academy of Sciences of Kazakhstan, Almaty, Kazakhstan

## Additional Information and Declarations

### Data Availability

The following information was supplied regarding data availability: The 3D CT scan data and reconstructed models of fossil giant salamander vertebrae (Cryptobranchidae, Limnospondylus ajimuensis) from the Pliocene Tsubusagawa Formation, Ajimu, Oita Prefecture, Japan (raw CT image stacks and derived surface models for three vertebrae (LBM0142000335–337)) are available at MorphoSource: - ID: 000785359 (10.17602/M2/M785359): https://www.morphosource.org/concern/media/000785359?locale=en
- ID: 000785313 (10.17602/M2/M785313): https://www.morphosource.org/concern/media/000785313?locale=en
- ID: 000785310 (10.17602/M2/M785310): https://www.morphosource.org/concern/media/000785310?locale=en
- ID: 000784248 (10.17602/M2/M784248): https://www.morphosource.org/concern/media/000784248?locale=en
- ID: 000784244 (10.17602/M2/M784244): https://www.morphosource.org/concern/media/000784244?locale=en
- ID: 000784240 (10.17602/M2/M784240): https://www.morphosource.org/concern/media/000784240?locale=en

### New Species Registration

The following information was supplied regarding the registration of a newly described species: Publication LSID: urn:lsid:zoobank.org:pub:D4514909-6F7F-4E66-9BA7-8A17113C6A4C. *Limnospondylus genus* LSID: urn:lsid:zoobank.org:act:2D9C2771-DEAE-4196-8052-F96F9B2012DF *Limnospondylus ajimuensis* LSID: urn:lsid:zoobank.org:act:0C82EBD9-DE30-45DA-B395-812F9F72F3D1
