# Supplementary figures and images for: A new genus of giant salamander (Urodela, Cryptobranchidae) from the Pliocene of Japan
Source: PeerJ. 2026 Jun 3;14:e21362. doi: 10.7717/peerj.21362 (PMC13242194; doi:10.7717/peerj.21362)

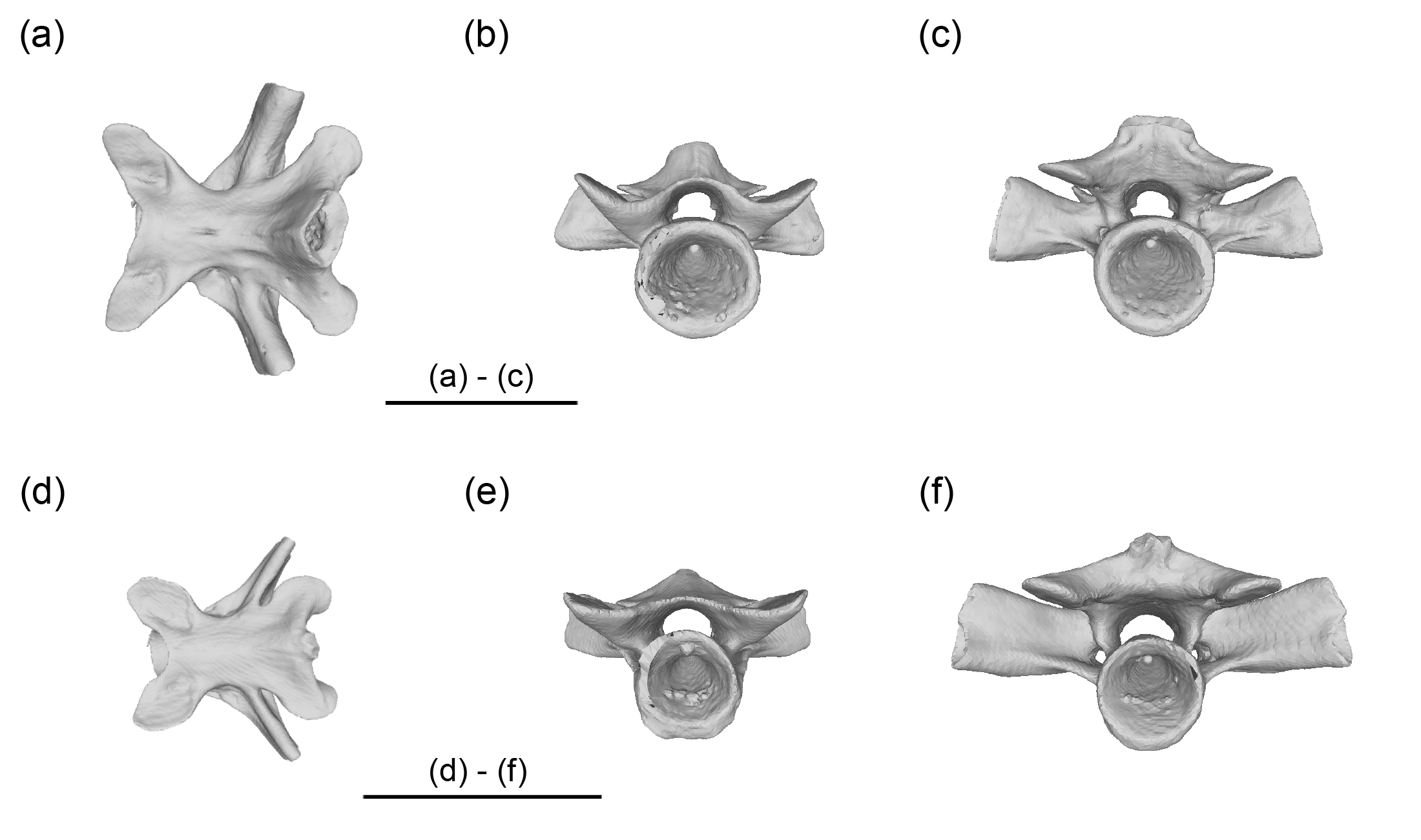

Supplement: Supplemental Information 3 [file peerj-14-21362-s003.png]
